# La cholecystite alithiasique, une manifestation inhabituelle au cours du purpura rhumatoïde de l'adulte

**DOI:** 10.11604/pamj.2015.22.48.7179

**Published:** 2015-09-18

**Authors:** Amira Atig, Neirouz Ghannouchi Jaafoura

**Affiliations:** 1 Service de Médecine Interne, CHU Farhat Hached, Sousse, Tunisie

**Keywords:** purpura rhumatoïde, cholecystite alithiasique, douleur abdominale, rheumatoid purpura, cholecystitis acalculous, abdominal pain

## Image en médecine

Un homme de 49 ans est hospitalisé pour un purpura vasculaire évoluant depuis 5 jours, associé à des douleurs abdominales maximales au niveau de l'hypochondre droit. A l'examen physique on note un purpura generalisé (A,B), ainsi qu'une sensibilité abdominale diffuse. Au bilan biologique, l'hémogramme montre une hyperleucocytose à polynucléaires neutrophiles sans atteinte des autres lignées, on note aussi une cholestase à 6 fois la normale et une cytolyse hépatique à 3 fois la normale. L’échographie abdominale montre des voies biliaires fines mais avec un épaississement vésiculaire sans mise en évidence de lithiase. Le scanner abdominal (C) confirme ces données qui sont en faveur d'une cholécystite alithiasique. La biopsie cutanée avec étude en immunofluorescence directe montrent une vascularite leucocytoclasique avec dépôts d'IgA permettant de retenir le diagnostic de purpura rhumatoïde. Les sérologies des hépatites B et C, le bilan des hépatopathies auto-immunes, la recherche d'anticorps anti-nucléaires et de cryoglobulines sont par ailleurs négatifs. Le patient a été traité par corticoïdes et inhibiteur de la pompe à protons avec amelioration des douleurs sans recours à la chirurgie. L'atteinte digestive au cours du purpura rhumatoïde peut siéger tout au long du tractus digestif. la cholécystite alithiasique est rare et elle est exceptionnellement révélatrice. Le recours à une cholécystectomie est parfois inévitable et le diagnostic de la vascularite est posé sur la pièce opératoire. Les symptômes ne sont pas spécifiques mais l'association à un purpura vasculaire, comme dans notre cas, oriente le diagnostic et permet d’éviter la chirurgie.

**Figure 1:**
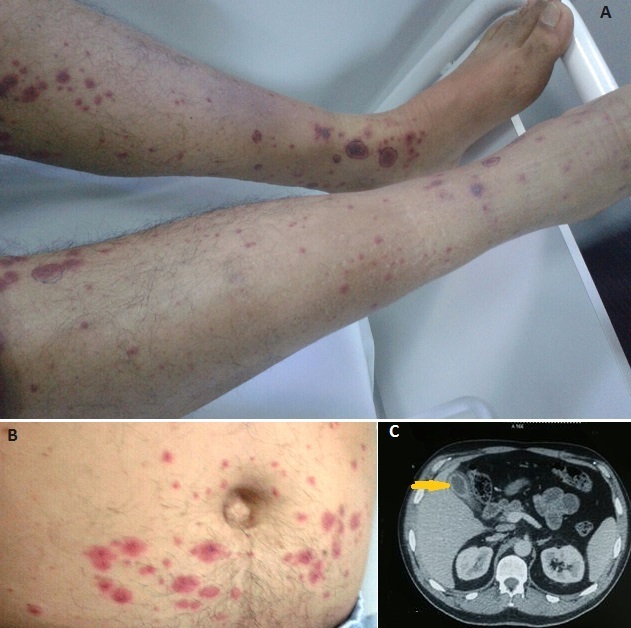
A) purpura vasculaire des membres inférieurs; B) purpura vasculaire du tronc; C) TDM abdominale: épaississement vésiculaire sans image de lithiase

